# NIA HEALTH DISPARITIES RESEARCH NETWORK: APPROACHES AND FINDINGS FROM GERIATRICS AND CLINICAL GERONTOLOGY

**DOI:** 10.1093/geroni/igz038.044

**Published:** 2019-11-08

**Authors:** Lyndon Joseph, CarlV Hill

**Affiliations:** 1 National Institute on Aging, Bethesda, Maryland, United States; 2 National Institute on Aging (NIA), Bethesda, Maryland, United States

## Abstract

Health disparities are differences in the incidence, prevalence and burden of diseases, mortality rates and causes of death that exist among population groups. Health disparities are associated with a broad, complex, and interrelated array of factors that influence health, accelerate aging and reduce life expectancy. NIA’s health disparities research goals are to understand environmental and sociocultural factors and related behavioral and biological mechanisms that diminish health and reduce life expectancy for vulnerable populations, explore the biological mechanisms through which disparities influence age-related change, and identify where disparities emerge in diagnosis, prognosis or treatment in geriatric conditions. Presentations will focus on whether structural-level discrimination may be a key factor in potentiating well known race-related health disparities especially those with an accelerated onset and may be associated with MRI-indicators of subclinical brain pathology; identifying biomarkers for early detection of cognitive and functional decline in high risk subpopulations and how ethnicity influences cerebral spinal fluid and imaging biomarkers link to early identification of cognitive and functional impairment ; effects of medication management and deprescribing among African American and Hispanic older adults with Alzheimer’s disease and related dementias and multiple chronic conditions; examine the use of multi-level factors and technology to overcome the barriers to urban-rural health disparities in managing many chronic diseases such as hepatitis C virus infection and delivery of appropriate medical services; and understanding the racial and ethnic differences in the link between environmental exposures and auto-immune comorbid asthma.

